# A Rare Insidious Case of Skin and Soft Tissue Infection Due to Mycobacterium abscessus: A Case Report

**DOI:** 10.7759/cureus.25725

**Published:** 2022-06-07

**Authors:** Diana D Cardenas, Tabassum Yasmin, Shadab Ahmed

**Affiliations:** 1 Internal Medicine, Nassau University Medical Center, East Meadow, USA; 2 Infectious Disease, Nassau University Medical Center, East Meadow, USA

**Keywords:** gene mutation, lipoabdominoplasty, macrolide resistance, skin and soft tissue infection, mycobacterium abscesses complex

## Abstract

*Mycobacterium abscessus* complex (MABc) is part of the rapid-growing non-tuberculous mycobacteria group that usually resides in natural water sources. When it affects humans, it can be highly resistant and difficult to manage. The most common presentation is localized, mainly in the lungs and soft tissue, but can be generalized in immunocompromised patients. Here we present a case report of a 40-year-old female with a chronic infection of the abdominal wall after abdominoplasty.

## Introduction

*Mycobacterium abscessus *complex (MABc) is one of the most important causes of nontuberculous mycobacteria (NTM) after *Mycobacterium avium *complex (MAC). This group of microorganisms is fast-growing and has shown difficulties in treatment due to the development of highly drug-resistant pathogens [1-2]. The broad spectrum of infection is mainly pulmonary and soft tissue disease, but reports of bone disease and disseminated disease are more common in immunocompromised individuals. The non-pulmonary infections have been associated with postsurgical or post-injection wounds with a rate of 43% of cases, localized community-acquired wound infections with a rate of 23% of cases, and disseminated cutaneous infections with a rate of 20% of cases [3].

This article describes a case of chronic recurrent draining sinus infection located in the abdominal wall after a lipoabdominoplasty.

## Case presentation

A 40-year-old female came to our facility due to chronic recurrent draining sinus on the abdomen wall. The patient underwent a lipoabdominoplasty in the Dominican Republic three months before hospitalization. The patient had a course of antibiotics with ertapenem and piperacillin/tazobactam in another facility, which was unsuccessful in clearing the infection. On the day of admission to our facility, the patient had recurrent fevers and generalized abdominal pain. Laboratory workup demonstrated normal leukocyte count, microcytic-hypochromic anemia, preserved renal function, elevated erythrocyte sedimentation rate, and normal C-reactive protein (Table 1).

**Table 1:** Test results of the patient during the first admission to our facility BUN: blood urea nitrogen; ESR: erythrocyte sedimentation rate; CRP: C-reactive protein

Blood test	Results	Reference value	Units of measurement
Leukocyte	7280	4500-11000	mm^3^
Hemoglobin	10.4	12.1-15.5	g/dL
Hematocrit	34.7	37-47	%
BUN	13	6-24	mg/dL
Creatinine	0.7	0.6-1.1	mg/dL
ESR	47	0-22	mm/Hr
CRP	0.9	0.8-1.0	mg/dL

**Figure 1:**
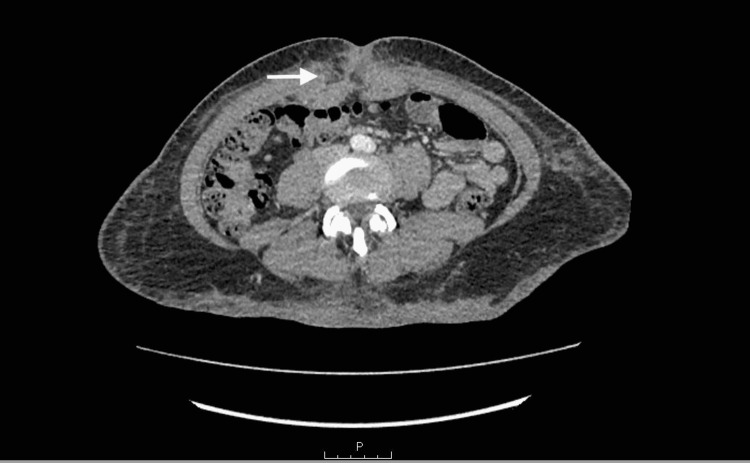
CT abdomen and pelvis with IV contrast The arrow shows diffuse abdominal wall subcutaneous stranding with no abdominal wall abscess.

The computed tomography (CT) of the abdomen and pelvis with contrast showed diffuse abdominal wall subcutaneous stranding without abdominal wall abscess (Figure 1).

The patient received ceftriaxone 2 g intravenously (IV) daily and had incision and drainage of the postoperative wound with debridement of 224 cm^2^ sinus tract of the abdomen. The wound vac was applied twice on this admission. Tissue specimen was sent for anaerobic, aerobic, fungal cultures, and acid-fast bacilli (AFB) and tissue for pathology. After six days of hospital course, the patient was discharged empirically on amoxicillin and clavulanic acid with an outpatient follow-up in the infectious disease clinic. 

**Figure 2:**
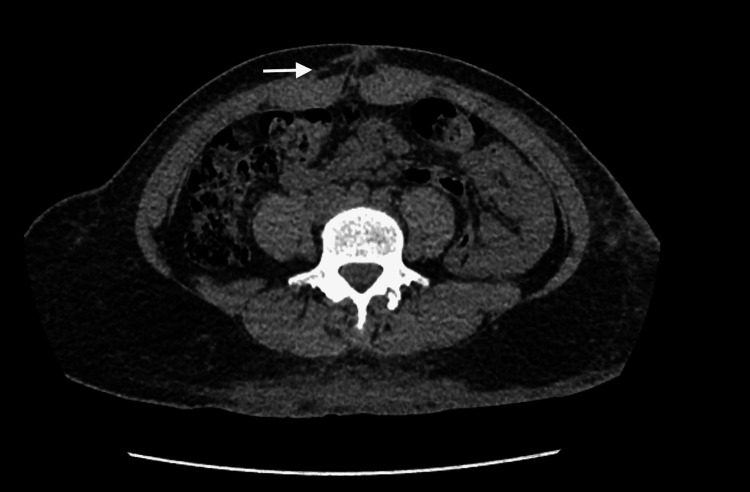
CT abdomen and pelvis The arrow shows abdominal wall subcutaneous stranding is unchanged no evidence for abdominal wall abscess.

During a clinic visit, the cultures results of the tissue sample came back positive for AFB *Mycobacterium abscesses *complex*, *but no susceptibilities were available at this point. The antibiotics were changed to linezolid 600 mg orally every 12 hours and clarithromycin 500 mg every 12 hours. The patient did not improve and after one month was readmitted to our facility. At this admission, CT abdomen/pelvis without contrast demonstrated unchanged abdominal wall subcutaneous stranding with no evidence for abdominal wall abscess (Figure 2).

The patient received meropenem 1 g IV every eight hours, amikacin 700mg IV daily, and continued clarithromycin 500 mg every 12 hours. The patient was discharged with IV meropenem, amikacin, and oral clarithromycin. The final results of the tissue sample demonstrated susceptibility to tigecycline, amikacin, and clarithromycin (Table 2). Antibiotics were changed, and the patient received omadacycline 300 mg daily, amikacin 900 mg IV daily, and oral clarithromycin 500 mg daily for six months.

**Table 2:** Antibiotic susceptibility of Mycobacterium abscessus complex of the tissue sample obtained in the incision and drainage S: susceptible; I: intermediate; R: resistant This assay detects inducible resistance to macrolide due to erm gene by extended 14 days incubation for clarithromycin

Antibiotic	Results	Unit of Measurement
Amikacin	16 S	mcg/mL
Cefoxitin	32 I	mcg/mL
Ciprofloxacin	>4 R	mcg/mL
Clarithromycin	1 S	mcg/mL
Doxycycline	>16 R	mcg/mL
Imipenem	16 I	mcg/mL
Linezolid	32 R	mcg/mL
Minocycline	>8 R	mcg/mL
Moxifloxacin	>8 R	mcg/mL
Tigecycline	0.5 S	mcg/mL
Trimethoprim/Sulfamethoxazole	>8/152 R	mcg/mL

## Discussion

*Mycobacterium abscessus *complex is classified as a rapid-growing mycobacterium (RGM), non-tuberculosis pathogen, and by far the most pathogenic of the RGM group [1,2]. Normally, this type of microorganism is found in natural and drinking water sources, sewage water, household plumbing, hospital wastewater, and soil and is non-pathogenic in most cases [4,5]. In the past, MABc was only reported in immunosuppressed. Lately, the number of cases has increased, and the population that has been affected is immunocompetent individuals [6]. Pre-existing conditions, such as cystic fibrosis and HIV, are more related to the pulmonary and disseminated presentation; on the other hand, soft tissue infections are linked to healthcare-associated infections [5].

MABc can be divided into three subspecies: Mycobacterium (*M.) abscessus *subspecies *abscessus *(45-65% cases reported), *M. abscessus *subspecies *massiliense* (20-55% cases reported), and *M. abscessus *subspecies *bolletii* (1-18% cases reported) [2].

Skin and soft tissue infections with MABc are related to surgical, plastic procedures, trauma, transplantation, and cancer, but nowadays, there are cases related to simple cosmetic procedures, such as pedicures, tattooing mesotherapy, and body piercing [4,7,8]. The presentation can be an abscess formation, chronic indolent ulcers, or disseminated disease [4,9]. Our patient had a surgical procedure that most likely put her at risk of developing this type of infection. Some hypotheses explain the potential causes of wound infection after cosmetic and surgical procedures. Open wounds can be contaminated with Gentian violet, antiseptic solutions, the use of tap water for postoperative irrigation or to clear surgical instruments, reuse of liposuction catheters, and the inexistence of autoclaving in those facilities [5,10].

According to the guidelines of the Infectious Diseases Society of America, the treatment is based on a combination therapy of antibiotics with surgical debridement. This type of bacteria usually is susceptible to macrolides, amikacin, cefoxitin, linezolid, and imipenem [2-5,11]. Clarithromycin has been demonstrated to be the drug of choice in the treatment of MABc infections. Treatment has been demonstrated to be difficult due to the high resistance; however, the extrapulmonary infection has better outcomes after four to six months of treatment, whereas pulmonary infection has high failure rates, even after more than 12 to 24 months of therapy [2,6-9].

There have been several described mechanisms of resistance in the MABc organism that can be intrinsic and extrinsic resistance [12]; wax cell barrier with high levels of lipids and biofilm formation decreases the penetration of antibiotics, and the formation of gene mutations, *erm(41) and rrl, *which confer macrolide resistance, *rrs* gene mutation with amikacin resistance, and *gyrA* and *gyrB* genes with quinolone resistance. The form to test the presence of resistant microorganisms is by performing a prolonged incubation period of 14 days. Genetic studies suggest that the three subspecies differ in specific *erm(41)* mutations and intrinsic clarithromycin susceptibility patterns. The strains that have intrinsic resistance to macrolides are *M. abscessus *subspecies* abscessus* and *M. abscessus *subspecies* bolletii*, because they possess a full-length and functional *erm(41)* gene. *M. abscessus *subsecies* massiliense* is more susceptible to macrolides due to a truncated, non-functional *erm(41)* gene but may acquire inducible macrolide resistance with antibiotic exposure [13-14].

Sfeir et al. describe risk factors for treatment failure, disseminated infection, resistance to clarithromycin, IV amikacin treatment receipt, acute kidney injury, presence of prosthetic device after prior transplantation, and immunosuppressive therapy [9]. The patient does not meet these risk factors but failed treatment with linezolid and clarithromycin due to linezolid resistance. The patient did respond well to omadacycline, amikacin, and clarithromycin.

## Conclusions

In conclusion, for a patient that reports chronic skin infections after any surgical procedure or invasive procedure without any improvement in the clinic scenario with empiric treatment and with negative tissue cultures, AFB smear and mycobacterium culture must be done to rule out NTM infectious. Regarding treatment, there has not been much investigation in the field, but according to the guidelines, the treatment is a combination of surgical debridement and a combination of antibiotics. 

## References

[1] Strnad L, Winthrop KL (2018). Treatment of Mycobacterium abscessus complex. Semin Respir Crit Care Med.

[2] Jeong SH, Kim SY, Huh HJ (2017). Mycobacteriological characteristics and treatment outcomes in extrapulmonary Mycobacterium abscessus complex infections. Int J Infect Dis.

[3] Gonzalez-Santiago TM, Drage LA (2015). Nontuberculous mycobacteria: skin and soft tissue infections. Dermatol Clin.

[4] Moreno-Izquierdo C, Zurita J, Contreras-Yametti FI, Jara-Palacios MA (2020). Mycobacterium abscessus subspecies abscessus infection associated with cosmetic surgical procedures: cases series. IDCases.

[5] To K, Cao R, Yegiazaryan A, Owens J, Venketaraman V (2020). General overview of nontuberculous mycobacteria opportunistic pathogens: Mycobacterium avium and Mycobacterium abscessus. J Clin Med.

[6] Wentworth AB, Drage LA, Wengenack NL, Wilson JW, Lohse CM (2013). Increased incidence of cutaneous nontuberculous mycobacterial infection, 1980 to 2009: a population-based study. Mayo Clin Proc.

[7] Petrini B (2006). Mycobacterium abscessus: an emerging rapid-growing potential pathogen. APMIS.

[8] Uslan DZ, Kowalski TJ, Wengenack NL, Virk A, Wilson JW (2006). Skin and soft tissue infections due to rapidly growing mycobacteria: comparison of clinical features, treatment, and susceptibility. Arch Dermatol.

[9] Sfeir M, Walsh M, Rosa R (2018). Mycobacterium abscessus complex infections: a retrospective cohort study. Open Forum Infect Dis.

[10] Rubio M, March F, Garrigó M, Moreno C, Español M, Coll P (2015). Inducible and Acquired Clarithromycin Resistance in the Mycobacterium abscessus Complex. PLoS One.

[11] Hui SH, Noonan L, Chavada R (2015). Post liposuction Mycobacterium abscessus surgical site infection in a returned medical tourist complicated by a paradoxical reaction during treatment. Infect Dis Rep.

[12] Lee MC, Sun PL, Wu TL (2017). Antimicrobial resistance in Mycobacterium abscessus complex isolated from patients with skin and soft tissue infections at a tertiary teaching hospital in Taiwan. J Antimicrob Chemother.

[13] Furuya EY, Paez A, Srinivasan A (2008). Outbreak of Mycobacterium abscessus wound infections among "lipotourists" from the United States who underwent abdominoplasty in the Dominican Republic. Clin Infect Dis.

[14] Meir M, Barkan D (2020). Alternative and experimental therapies of Mycobacterium abscessus infections. Int J Mol Sci.

